# Supplementary material for: The target of rapamycin signaling pathway regulates vegetative development, aflatoxin biosynthesis, and pathogenicity in Aspergillus flavus
Source: eLife. 2024 Jul 11;12:RP89478. doi: 10.7554/eLife.89478 (PMC11239180; doi:10.7554/eLife.89478)
Supplement: Figure 2—figure supplement 1—source data 1. [file elife-89478-fig2-figsupp1-data1.zip › Title for source data files.docx]

**Title for source data files**

**Figure 2—figure supplement 1—source data 1**.Original file for the DNA gel electrophoresis analysis in Figure 2—figure supplement 1A (Δfkbp1).

**Figure 2—figure supplement 1—source data 2**.PDF containing Figure 2—figure supplement 1A and original images of the DNA gel electrophoresis analysis (Δfkbp1) with highlighted bands and sample labels.

**Figure 2—figure supplement 1—source data 3**.Original file for the DNA gel electrophoresis analysis in Figure 2—figure supplement 1A (Δfkbp2).

**Figure 2—figure supplement 1—source data 4**.PDF containing Figure 2—figure supplement 1A and original images of the DNA gel electrophoresis analysis (Δfkbp2) with highlighted bands and sample labels.

**Figure 2—figure supplement 1—source data 5**.Original file for the DNA gel electrophoresis analysis in Figure 2—figure supplement 1A (Δfkbp3).

**Figure 2—figure supplement 1—source data 6**.PDF containing Figure 2—figure supplement 1A and original images of the DNA gel electrophoresis analysis (Δfkbp3) with highlighted bands and sample labels.

**Figure 2—figure supplement 1—source data 7**.Original file for the DNA gel electrophoresis analysis in Figure 2—figure supplement 1A (Δfkbp4).

**Figure 2—figure supplement 1—source data** **8**.PDF containing Figure 2—figure supplement 1A and original images of the DNA gel electrophoresis analysis (Δfkbp4) with highlighted bands and sample labels.

**Figure 2—figure supplement 1—source data 9**.Original file for the DNA gel electrophoresis analysis in Figure 2—figure supplement 1C (^xylP^torA).

**Figure 2—figure supplement 1—source data 10**.PDF containing Figure 2—figure supplement 1C and original images of the DNA gel electrophoresis analysis (^xylP^torA) with highlighted bands and sample labels.

**Figure 2—figure supplement 1—source data 11**.Original file for the DNA gel electrophoresis analysis in Figure 2—figure supplement 1D (OE::tapA).

**Figure 2—figure supplement 1—source data 12**.PDF containing Figure 2—figure supplement 1D and original images of the DNA gel electrophoresis analysis (OE::tapA) with highlighted bands and sample labels.

**Figure 2—figure supplement 1—source data 13**.Original file for the DNA gel electrophoresis analysis in Figure 2—figure supplement 1E (ΔtipA).

**Figure 2—figure supplement 1—source data 14**.PDF containing Figure 2—figure supplement 1E and original images of the DNA gel electrophoresis analysis (ΔtipA) with highlighted bands and sample labels.

**Figure 2—figure supplement 1—source data 15**.Original file for the DNA gel electrophoresis analysis in Figure 2—figure supplement 1F (Δsch9).

**Figure 2—figure supplement 1—source data 16**.PDF containing Figure 2—figure supplement 1F and original images of the DNA gel electrophoresis analysis (Δsch9) with highlighted bands and sample labels.

**Figure 2—figure supplement 1—source data 17**.Original file for the DNA gel electrophoresis analysis in Figure 2—figure supplement 1F (sch9^ΔC2^).

**Figure 2—figure supplement 1—source data 18**.PDF containing Figure 2—figure supplement 1F and original images of the DNA gel electrophoresis analysis (sch9^ΔC2^) with highlighted bands and sample labels.

**Figure 2—figure supplement 1—source data 19**.Original file for the DNA gel electrophoresis analysis in Figure 2—figure supplement 1F (sch9^ΔS_TKc^).

**Figure 2—figure supplement 1—source data 20**.PDF containing Figure 2—figure supplement 1F and original images of the DNA gel electrophoresis analysis (sch9^ΔS_TKc^) with highlighted bands and sample labels.

**Figure 2—figure supplement 1—source data 21**.Original file for the DNA gel electrophoresis analysis in Figure 2—figure supplement 1F (sch9^ΔS_TK_X^).

**Figure 2—figure supplement 1—source data 22**.PDF containing Figure 2—figure supplement 1F and original images of the DNA gel electrophoresis analysis (sch9^ΔS_TK_X^) with highlighted bands and sample labels.

**Figure 2—figure supplement 1—source data 23**.Original file for the DNA gel electrophoresis analysis in Figure 2—figure supplement 1G (ΔsitA and ΔsitA-Com).

**Figure 2—figure supplement 1—source data 24**.PDF containing Figure 2—figure supplement 1G and original images of the DNA gel electrophoresis analysis (ΔsitA and ΔsitA-Com) with highlighted bands and sample labels.

**Figure 2—figure supplement 1—source data 25**.Original file for the DNA gel electrophoresis analysis in Figure 2—figure supplement 1H (Δppg1 and Δppg1-Com).

**Figure 2—figure supplement 1—source data 26**.PDF containing Figure 2—figure supplement 1H and original images of the DNA gel electrophoresis analysis (Δppg1 and Δppg1-Com) with highlighted bands and sample labels.

**Figure 2—figure supplement 1—source data 27**.Original file for the DNA gel electrophoresis analysis in Figure 2—figure supplement 1I (ΔsitA/ppg1).

**Figure 2—figure supplement 1—source data 28**.PDF containing Figure 2—figure supplement 1I and original images of the DNA gel electrophoresis analysis (ΔsitA/ppg1) with highlighted bands and sample labels.

**Figure 2—figure supplement 1—source data 29**.Original file for the DNA gel electrophoresis analysis in Figure 2—figure supplement 1J (Δnem1).

**Figure 2—figure supplement 1—source data 30**.PDF containing Figure 2—figure supplement 1J and original images of the DNA gel electrophoresis analysis (Δnem1) with highlighted bands and sample labels.

**Figure 2—figure supplement 1—source data 31**.Original file for the DNA gel electrophoresis analysis in Figure 2—figure supplement 1K (Δspo7).

**Figure 2—figure supplement 1—source data 32**.PDF containing Figure 2—figure supplement 1K and original images of the DNA gel electrophoresis analysis (Δspo7) with highlighted bands and sample labels.
