# Supplementary figures and images for: The target of rapamycin signaling pathway regulates vegetative development, aflatoxin biosynthesis, and pathogenicity in Aspergillus flavus
Source: eLife. 2024 Jul 11;12:RP89478. doi: 10.7554/eLife.89478 (PMC11239180; doi:10.7554/eLife.89478)

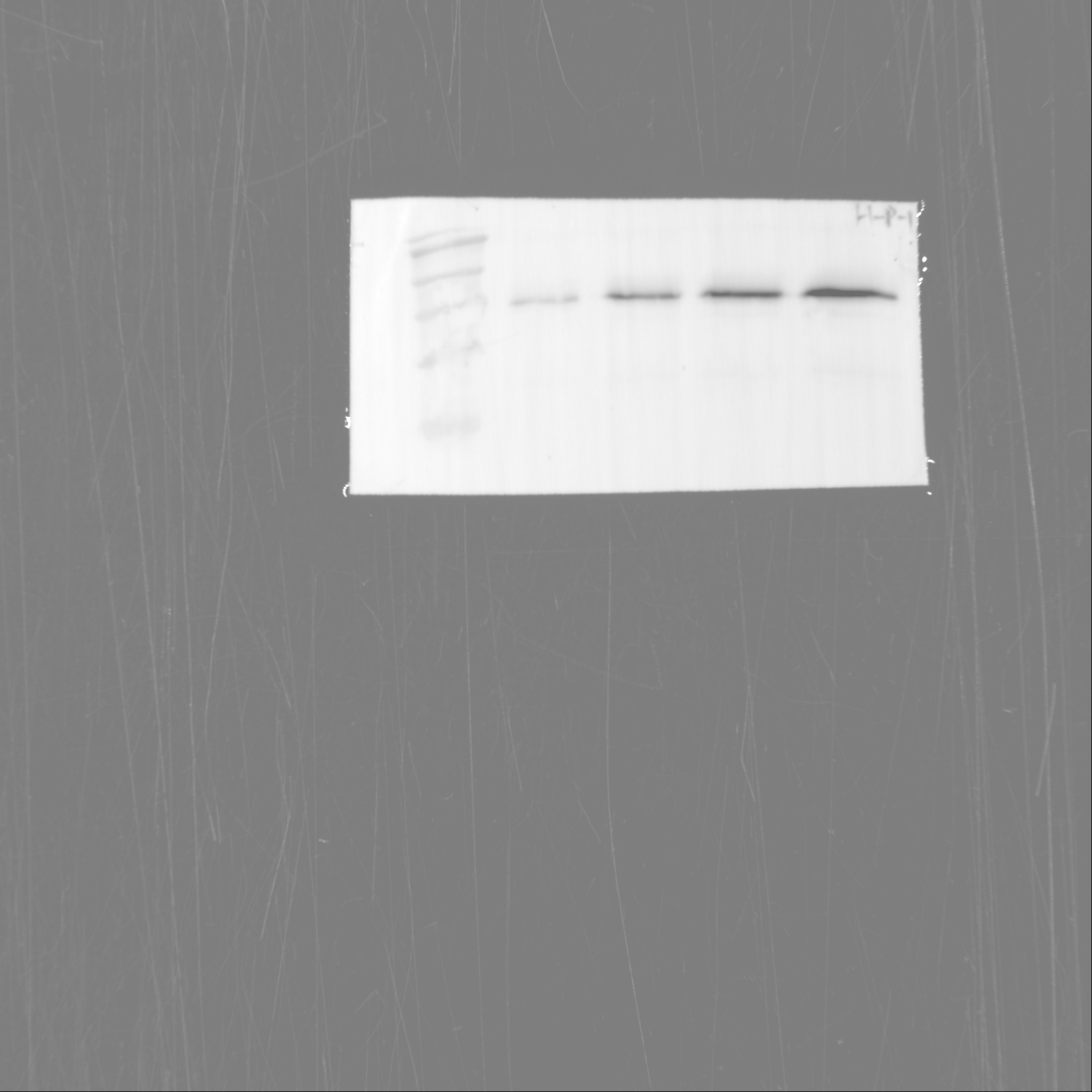

Supplement: Figure 4—source data 1. [file elife-89478-fig4-data1.zip › Figure 4 -source data 1/Figure 4 -source data 1.tif]

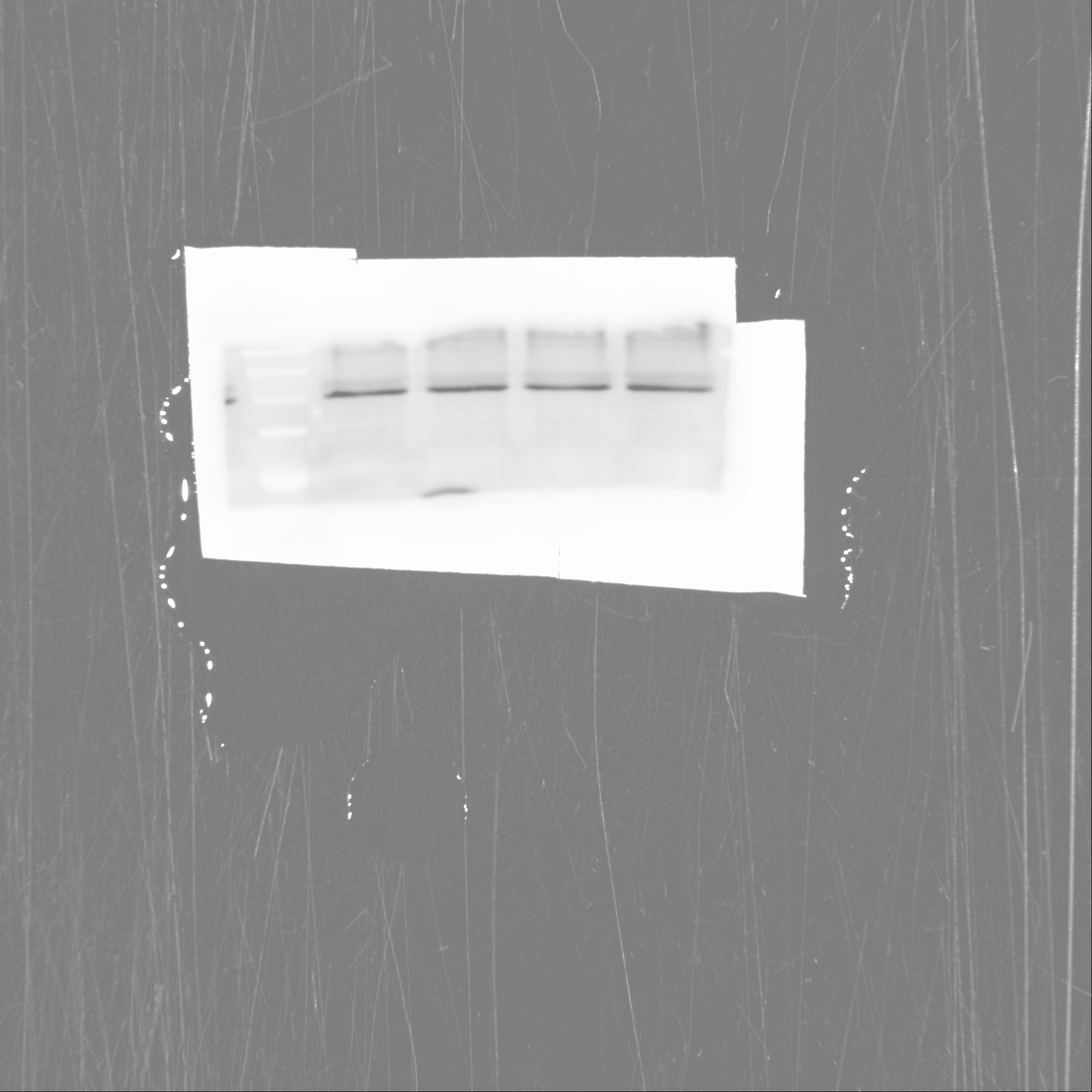

Supplement: Figure 4—source data 2. [file elife-89478-fig4-data2.zip › Figure 4 -source data 2/Figure 4 -source data 2.tif]

**E**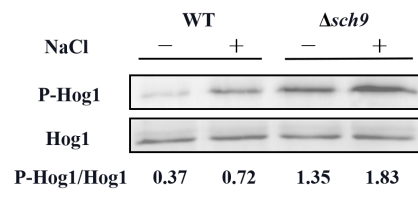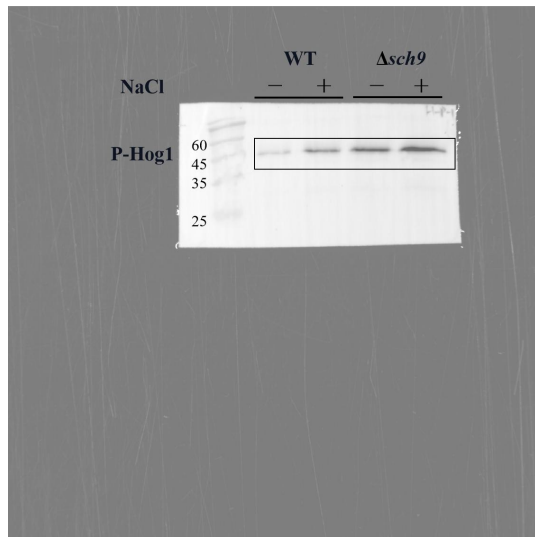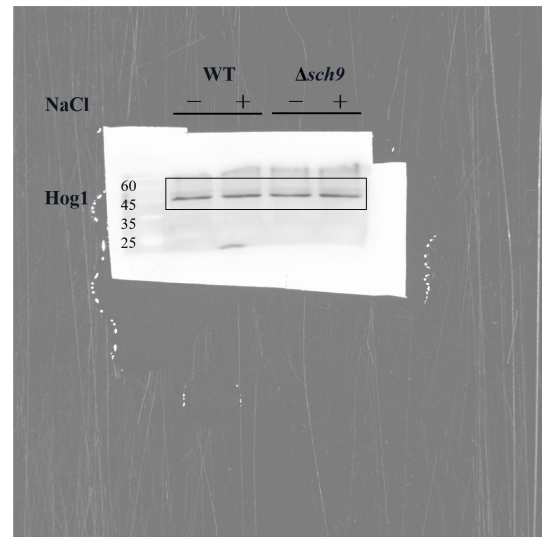**Figure 4**

Supplement: Figure 4—source data 3. [file elife-89478-fig4-data3.zip › Figure 4 -source data 3/Figure 4 -source data 3.pdf]

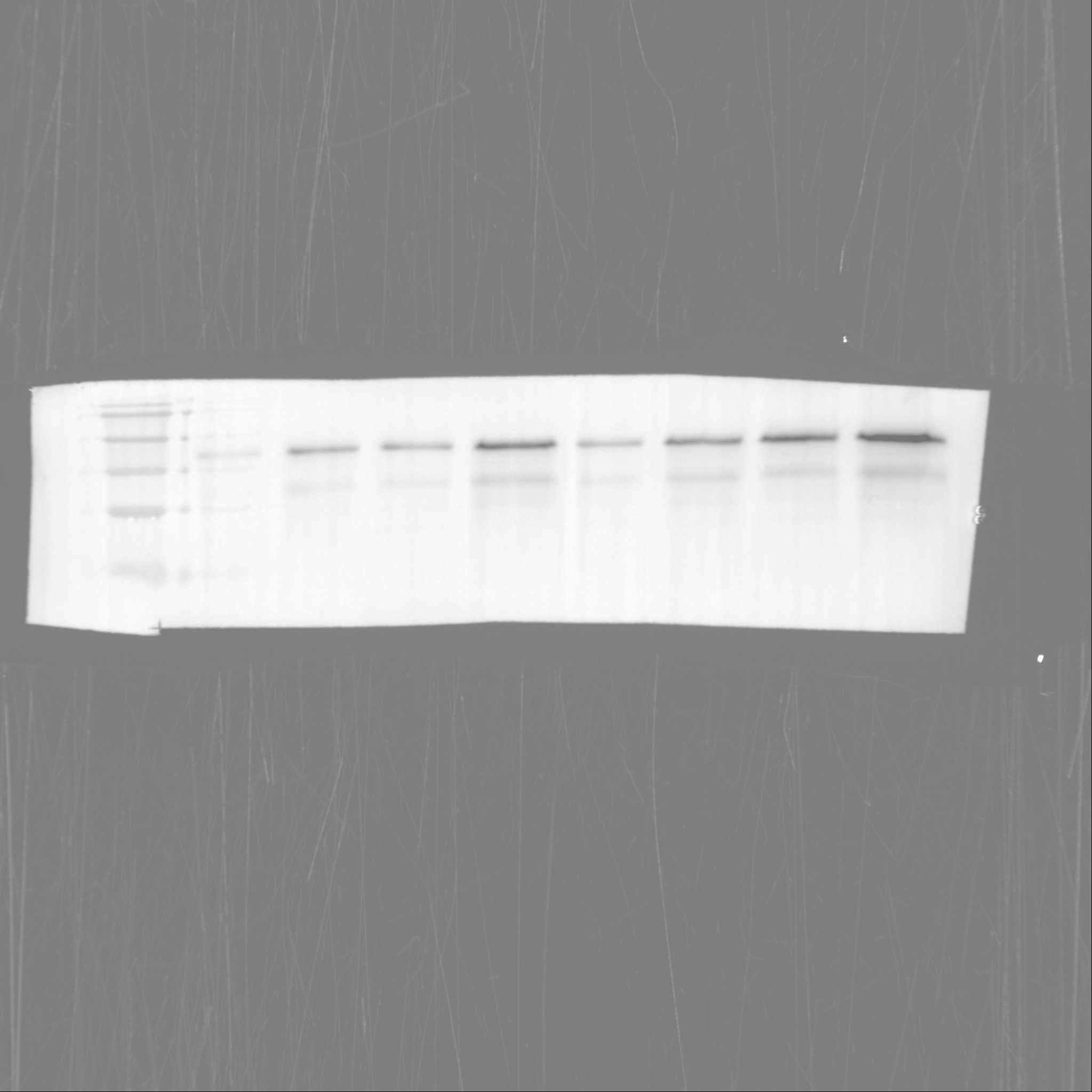

Supplement: Figure 9—source data 1. [file elife-89478-fig9-data1.zip › Figure 9 -source data 1/Figure 9 -source data 1.tif]

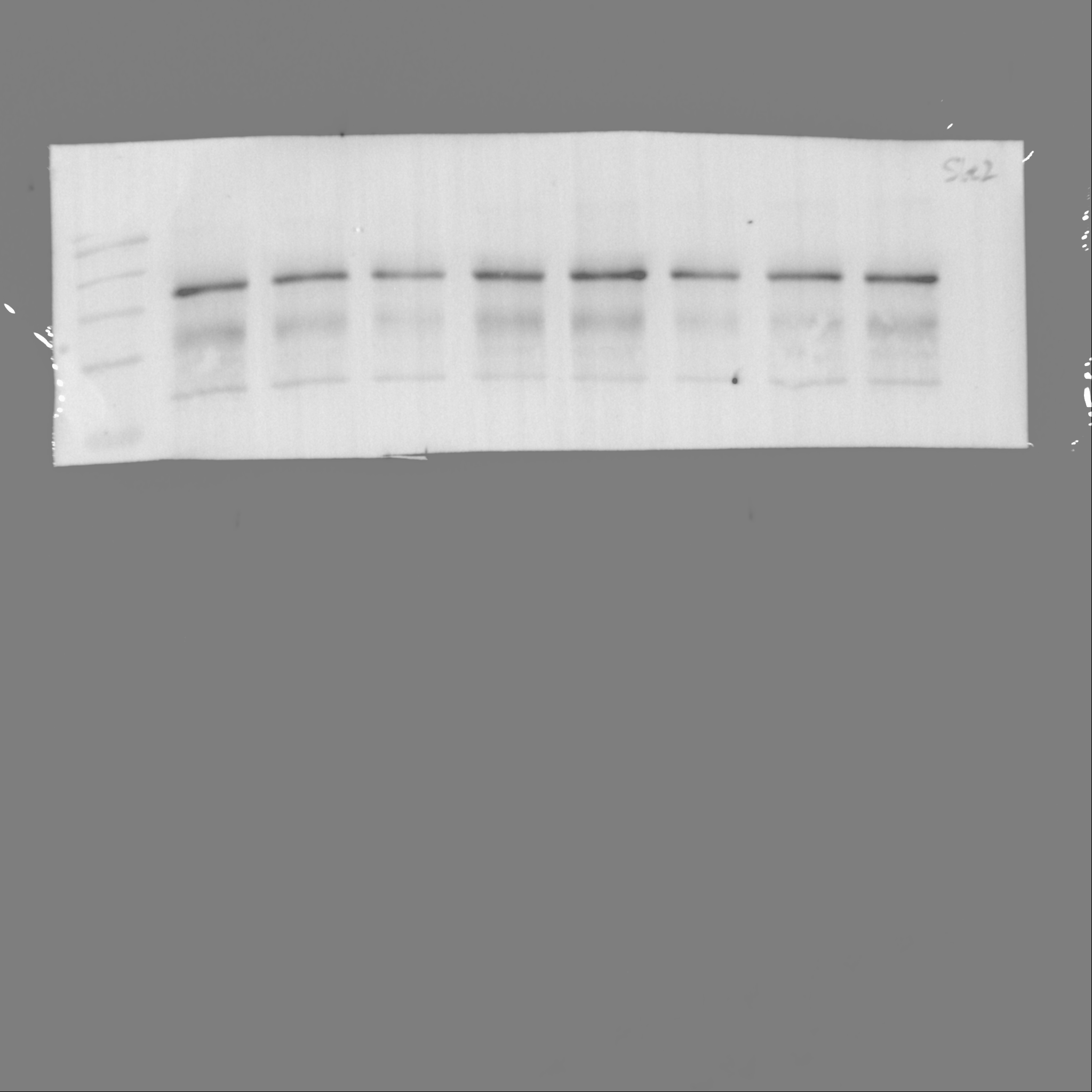

Supplement: Figure 9—source data 2. [file elife-89478-fig9-data2.zip › Figure 9 -source data 2/Figure 9 -source data 2.tif]

**D**

|             | WT   |      | $\Delta sitA$ |      | $\Delta ppG1$ |      | $\Delta sitA\Delta ppG1$ |      |
|-------------|------|------|---------------|------|---------------|------|--------------------------|------|
| CFW         | -    | +    | -             | +    | -             | +    | -                        | +    |
| P-Slt2      |      |      |               |      |               |      |                          |      |
| Slt2        |      |      |               |      |               |      |                          |      |
| P-Slt2/Slt2 | 0.13 | 0.44 | 0.5           | 0.72 | 0.24          | 0.61 | 0.86                     | 1.05 |

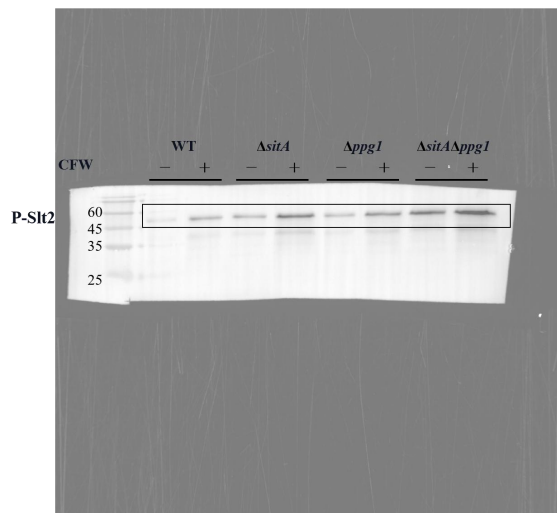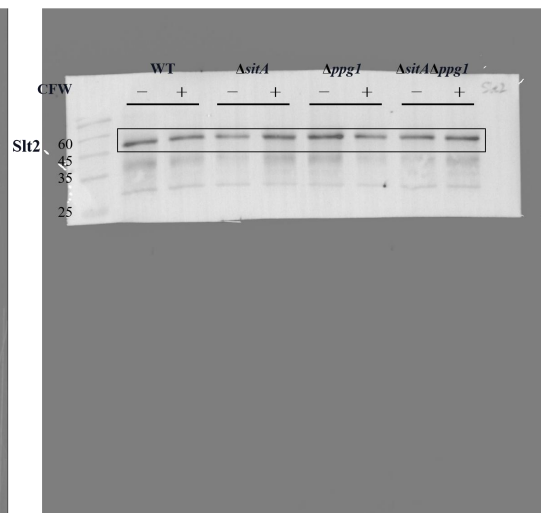

**Figure 9**

Supplement: Figure 9—source data 3. [file elife-89478-fig9-data3.zip › Figure 9 -source data 3/Figure 9 -source data 3.pdf]
