# Supplementary material for: The target of rapamycin signaling pathway regulates vegetative development, aflatoxin biosynthesis, and pathogenicity in Aspergillus flavus
Source: eLife. 2024 Jul 11;12:RP89478. doi: 10.7554/eLife.89478 (PMC11239180; doi:10.7554/eLife.89478)
Supplement: Supplementary file 1. [file elife-89478-supp1.docx]

| **Supplementary file 1a**. **Strains used in this study.** | | |
| --- | --- | --- |
| Strain | Genotype description | References |
| *A. flavus* PTS | Δ*ku70*; Δ*niaD*; Δ*pyrG* | Chang et al, 2010 |
| *A. flavus* wild-type | Δ*ku70*; Δ*niaD*; Δ*pyrG*::*pyrG* | Saved in our lab |
| *A. flavus* Δ*fkbp1* | Δ*ku70*; Δ*niaD*; Δ*fkbp1*::*pyrG* | This study |
| *A. flavus* Δ*fkbp2* | Δ*ku70*; Δ*niaD*; Δ*fkbp2*::*pyrG* | This study |
| *A. flavus* Δ*fkbp3* | Δ*ku70*; Δ*niaD*; Δ*fkbp3*::*pyrG* | This study |
| 1. *flavus* Δ*fkbp4* | Δ*ku70*; Δ*niaD*; Δ*fkbp4*::*pyrG* | This study |
| *A. flavus fkbp3* ^K5A^ | Δ*ku70*; Δ*niaD*; *fkbp3* ^K5A^::*pyrG* | This study |
| *A. flavus fkbp3* ^K19A^ | Δ*ku70*; Δ*niaD*; *fkbp3* ^K19A^::*pyrG* | This study |
| *A. flavus fkbp3* ^K40A^ | Δ*ku70*; Δ*niaD*; *fkbp3* ^K40A^::*pyrG* | This study |
| *A. flavus fkbp3* ^K42A^ | Δ*ku70*; Δ*niaD*; *fkbp3* ^K42A^::*pyrG* | This study |
| *A. flavus fkbp3* ^K55A^ | Δ*ku70*; Δ*niaD*; *fkbp3* ^K55A^::*pyrG* | This study |
| *A. flavus fkbp3* ^K65A^ | Δ*ku70*; Δ*niaD*; *fkbp3* ^K65A^::*pyrG* | This study |
| *A. flavus ^xylP^torA* | Δ*ku70*; Δ*niaD*; *xylP(torA)*::*pyrG* | This study |
| *A. flavus* Δ*sch9* | Δ*ku70*; Δ*niaD*; Δ*sch9*::*pyrG* | This study |
| *A. flavus sch9* ^ΔC2^ | Δ*ku70*; Δ*niaD*; *sch9* ^ΔC2^::*pyrG* | This study |
| *A. flavus sch9* ^ΔS_TKc^ | Δ*ku70*; Δ*niaD*; *sch9* ^ΔS_TKc^ ::*pyrG* | This study |
| *A. flavus sch9* ^ΔS_TK_X^ | Δ*ku70*; Δ*niaD*; *sch9* ^ΔS_TK_X^::*pyrG* | This study |
| *A. flavus sch9* ^K340A^ | Δ*ku70*; Δ*niaD*; *sch9* ^K340A^::*pyrG* | This study |
| *A. flavus OE*::*tapA* | Δ*ku70*; Δ*niaD*; *gpdA(tapA):: pyrG* | This study |
| *A. flavus* Δ*tipA* | Δ*ku70*; Δ*niaD*; Δ*tipA*::*pyrG* | This study |
| *A. flavus* Δ*sitA* | Δ*ku70*; Δ*niaD*; Δ*sitA*::*pyrG* | This study |
| *A. flavus* Δ*ppg1* | Δ*ku70*; Δ*niaD*; Δ*ppg1*::*pyrG* | This study |
| *A. flavus* Δ*sitA/ppg1* | Δ*ku70*; Δ*niaD*; Δ*ppg1*::*pyrG*, Δ*sitA*::*ptr* | This study |
| *A. flavus* Δ*sitA-Com* | *∆ku70*; *∆niad*; *∆sitA*::*sitA*::*pyrG* | This study |
| *A. flavus* Δ*ppg1-Com* | *∆ku70*; *∆niad*; *∆ppg1*::*ppg1*::*pyrG* | This study |
| *A. flavus* Δ*nem1* | Δ*ku70*; Δ*niaD*; Δ*nem1*::*pyrG* | This study |
| *A. flavus* Δ*spo7* | Δ*ku70*; Δ*niaD*; Δ*spo7*::*pyrG* | This study |

| **Supplementary file 1b. Primers used in this study.** | | |
| --- | --- | --- |
| Primer | Sequence（5’-3’） | Application |
| *fkbp1* L-F | CAATAGCAGCAACAGCCTCA | *fkbp1*deletion |
| *fkbp1* L-R | GGGTGAAGAGCATTGTTTGAGGCGCTGGTGGAGTTCGTATCGA |  |
| *fkbp1* R-F | GCATCAGTGCCTCCTCTCAGACCATCCACTCCTCAATAGACT |  |
| *fkbp1* R-R | CGAGGCATGATGATATCGAC |  |
| *fkbp1* C-F | ATGGTGATGGAGTTGAGTTG |  |
| *fkbp1* C-R | CATTGCGCGCATATCTCAC |  |
| *fkbp1* O-F | CAAGGTCTCCATCCACTACA |  |
| *fkbp1* O-R | TGATAATGCGATGCCACTAG |  |
| *fkbp*2 L-F | AGTGGAGTGCAAGGACCTT | *fkbp2* deletion |
| *fkbp*2 L-R | GGGTGAAGAGCATTGTTTGAGGCGCGTGGTTGAGTTAATTGAG |  |
| *fkbp*2 R-F | GCATCAGTGCCTCCTCTCAGACCCACGGTGTTGCTTGGTACT |  |
| *fkbp*2 R-R | TACAGCAAGTACCGAGTGAT |  |
| *fkbp*2 C-F | ATGGTCGGTTCCATGTTCGG |  |
| *fkbp*2 C-R | TAGTCTGCGCATAATGTTGG |  |
| *fkbp*2 O-F | ATGCGTTTCTCAATCTTCTC |  |
| *fkbp*2 O-R | CTCATCCTTCGAAACACCAT |  |
| *fkbp*3 L-F | CATCCGTTGTTTATGCTCTG | *fkbp3 deletion* |
| *fkbp*3 L-R | GGGTGAAGAGCATTGTTTGAGGCGTCGGTACCTGGATTGTGTA |  |
| *fkbp*3 R-F | GCATCAGTGCCTCCTCTCAGACTCGAATGCCTAACCTCACCA |  |
| *fkbp*3 R-R | TCATTGAAGTCCTTACCTGG |  |
| *fkbp*3 C-F | CATTCAAGAGACCTTCCAGA |  |
| *fkbp*3 C-R | GTGAACAGGCACAGGTCCTA |  |
| *fkbp*3 O-F | ATGGGTGTCACTAAGACGCT |  |
| *fkbp*3 O-R | GCTTTGGCATCTCCTTGTTG |  |
| *fkbp*4 L-F | ATCTGATCCACCAGCCTCCA | *fkbp4* deletion |
| *fkbp*4 L-R | GGGTGAAGAGCATTGTTTGAGGCATCTGATCCACCAGCCTCCA |  |
| *fkbp*4 R-F | GCATCAGTGCCTCCTCTCAGACGTATCACGGTGGCAGTGTT |  |
| *fkbp*4 R-R | TCATCACAGCAGTAGGAGTC |  |
| *fkbp*4 C-F | CCACGTAGATAGGTGATGCA |  |
| *fkbp*4 C-R | AGCTTGAAGGGTCGGAACTA |  |
| *fkbp*4 O-F | ATGTCTGTCCAACCTGTCGC |  |
| *fkbp*4 O-R | TCACCACCAACGGCCATACC |  |
| K5A A-F | GAAAGGATAGGCTGACTCGC | *fkbp3* ^K5A^ site-specific mutagenesis  *fkbp3* ^K5A^ site-specific mutagenesis |
| K5A A-R | GGCTGCGATAAGCGTCGCAGTGACACCCATTTTGAG |  |
| K5A B-F | CTCAAAATGGGTGTCACTGCGACGCTTATCGCAGCC |  |
| K5A B-R | GGGTGAAGAGCATTGTTTGAGGCTTAGGGCTTTGGCATCTCCTTGTTG |  |
| K5A C-F | GCATCAGTGCCTCCTCTCAGACTCGAATGCCTAACCTCACCAAAGACC |  |
| K5A C-R | ATGAGGATACGAGGCTCGAG |  |
| K5A N-F | GCCAGACCATAGCATTCTAG |  |
| K5A N-R | TCGATGGTGTGAACAGGCAC |  |
| K19A A-R | GGCGACAGTTTCATTCGCCTTGGGGAAGTTGAC | *fkbp3* ^K19A^ site-specific mutagenesis |
| K19A B-F | TCAACTTCCCCAAGGCGAATGAAACTGTCGCC |  |
| K40A A-R | CATACTGTTTTCCCGCGTTATCAGGCTGGCTAGG | *fkbp3* ^K40A^ site-specific mutagenesis |
| K40A B-F | GACCCTAGCCAGCCTGATAACGCGGGAAAACAG |  |
| K42A A-R | CCAACATACTGTGCTCCCTTGTTATCAGGCTGGC | *fkbp3* ^K42A^ site-specific mutagenesis |
| K42A B-F | CCAGCCTGATAACAAGGGAGCACAGTATGTTGG |  |
| K55A A-R | TCCTGTGCCAATAGCCACCGCCAAGGGCTGAC | *fkbp3* ^K55A^ site-specific mutagenesis |
| K55A B-F | GGGTCAGCCCTTGGCGGTGGCTATTGGCACA |  |
| K65A A-R | CGGCACACAAACCTGCAATAACACGTCCTGTGCC | *fkbp3* ^K65A^ site-specific mutagenesis |
| K65A B-F | GGCACAGGACGTGTTATTGCAGGTTTGTGTGCCG |  |
| *torA* X-L-F | CTTCGAGCTATTGGACCTGCCCT | *^xylP^torA* mutant |
| *torA* X-L-R | GGGTGAAGAGCATTGTTTGAGGCGTGGTCACCATAGGATCGA |  |
| *torA* X-R-F | CATTCATCGACTCGAAGAACCAACATGGCGCAAGCAGGTCCTATTAC |  |
| *torA* X-R-R | GGATAGTAGGCTTGATTTGTGGG |  |
| *torA* X-C-F | GTACTGGGTCACTCCTATCC |  |
| *torA* X-C-R | TGACAACATGCTGATGCACTCG |  |
| *torA* Q-F | CTGCTGGACTGAGTTGTATG | qRT-PCR for *torA* gene |
| *torA* Q-R | TCGTGCTCCATAAACTCAGC |  |
| *sch9* L-F | GACATTCGAATGTTGTCACCGT | *sch9* deletion  *sch9* deletion |
| *sch9* L-R | GGGTGAAGAGCATTGTTTGAGGCTACCAAGCGCAGGTGGAGGGAT |  |
| *sch9* R-F | GCATCAGTGCCTCCTCTCAGACTGATCCGATCTAGTATATGC |  |
| *sch9* R-R | GTTCCTTGATGCGAAGGAAGAG |  |
| *sch9* C-F | AACCAATAGTAGACCGAAGC |  |
| *sch9* C-R | CTTGATGGCAAGTGTCAAGG |  |
| *sch9* O-F | TCTACAGGACGACGACAATC |  |
| *sch9* O-R | ATAGACCTGACCGAATGTGC |  |
| *sch9* C2 A-F | CCGCTGGTCTAGAATGTATC | *sch9* C2 domain deletion |
| *sch9* C2 A-R | TGCGCCGCGAGCAGACAAGGGGAAGCCCTTGATCGGAGCAACTGGCGG |  |
| *sch9* C2 B-F | CCGCCAGTTGCTCCGATCAAGGGCTTCCCCTTGTCTGCTCGCGGCGCA |  |
| *sch9* C2 B-R | GGGTGAAGAGCATTGTTTGAGGCGGGCATTAAACTTCATAATTCAT |  |
| *sch9* C2 C-F | GCATCAGTGCCTCCTCTCAGACTGATCCGATCTAGTATATGCATTCTG |  |
| *sch9* C2 C-R | CAGTTGATGTGTGTGCCGTA |  |
| *sch9* C2 N-F | TCTACAGGACGACGACAATC |  |
| *sch9* C2 N-R | TCTTGAGCCCATACGTGTGG |  |
| *sch9* S_TKc A-R | GACAAAGCGTCCCAATCCACATCATGATCATCCGGGCCCACCTGTTTC | *sch9* S_TKc domain deletion |
| *sch9* S_TKc B-F | GAAACAGGTGGGCCCGGATGATCATGATGTGGATTGGGACGCTTTGTC |  |
| *sch9* S_TK_X A-R | CATATGATCTGTGGGTTCATGCTTGAAAAATGGGTGTTCCTTGAGTTC | *sch9* S_TK_X domain deletion |
| *sch9* S_TK_X B-F | GAACTCAAGGAACACCCATTTTTCAAGCATGAACCCACAGATCATATG |  |
| K340A A-F | GATAAGCGCCAGCCTTCCAT | *sch9* ^K340A^ site-specific mutagenesis |
| K340A A-R | CCTTCTTCGACAAAACTGCCATGGCATAAATTCGGC |  |
| K340A B-F | ACACGCCGAATTTATGCCATGGCAGTTTTGTCGAAG |  |
| K340A B-R | GGGTGAAGAGCATTGTTTGAGGCGGGCATTAAACTTCATAATTCATTT |  |
| K340A C-F | GCATCAGTGCCTCCTCTCAGACTGATCCGATCTAGTATATGCATTCTG |  |
| K340A C-R | GTAGAGGTTCCTTGATGCGA |  |
| K340A N-F | TATTGCTCTGCGTCTTCCCTGC |  |
| K340A N-R | CTTGATGGCAAGTGTCAAGG |  |
| *tapA* OE-L-F | GGTCCCAGTTCGGAACTAGC | *OE*::*tapA* mutant |
| *tapA* OE-L-R | AGGGTGAAGAGCATTGTTTGAGGCTGTAGTGGTTTAAGGTAGTA |  |
| *tapA* OE-R-F | CCAAGAACCTTTATTTCCCCTAAGATGGAACAACCCCAGAGTCTTCG |  |
| *tapA* OE-R-R | GCGTGGATGGTAAGGAAAGC |  |
| *tapA* OE-C-F | TAGTACAATTAGGCGAGAAG |  |
| *tapA* OE-C-R | TATAGATAGATGTCCTGTGGCG |  |
| *tapA* Q-F | GTATACTCACCAGACGTTTC | qRT-PCR for *tapA* gene |
| *tapA* Q-R | GTATTCCGACTGTTGAGCGT |  |
| *tipA* L-F | ACGAGGTCAATGGCGATTCA | *tipA* deletion  *tipA* deletion |
| *tipA* L-R | GGGTGAAGAGCATTGTTTGAGGCGAATAGATAGCGGAAAGTGC |  |
| *tipA* R-F | GCATCAGTGCCTCCTCTCAGACCGCTGTATCAAACTGAGGAG |  |
| *tipA* R-R | TATTGTGGCCATAGCAAGGC |  |
| *tipA* C-F | CTATGAGGAGTAAGCATGTG |  |
| *tipA* C-R | GTCCATGCGATGTTCAGCAT |  |
| *tipA* O-F | ATGTCTTCTGAGAACTCTCG |  |
| *tipA* O-R | CCTGTCGAACTGTCTCATAG |  |
| *sitA* L-F | CCTTGGTGGCTTTATCCG | *sitA* deletion |
| *sitA* L-R | GGGTGAAGAGCATTGTTTGAGGCAGGGTCAGAACGGTCA |  |
| *sitA* R-F | GCATCAGTGCCTCCTCTCAGACGTTCGGAGAAACCGTTCA |  |
| *sitA* R-R | TCCATTAGAACACGAAAGAC |  |
| *sitA* C-F | TAAGGGTGTGGTGGTCACGG |  |
| *sitA* C-R | GGCCTGGTTCAAGATTACAT |  |
| *sitA* O-F | CAAGTATCTTTCAGAGCAGCAT |  |
| *sitA* O-R | TTATCTCCAAACAGCCAAC |  |
| *ppg1* L-F | TAGGCTCCACTCCAAATA | *ppg1* deletion |
| *ppg1* L-R | GGGTGAAGAGCATTGTTTGAGGCGTCTGCCAGCAACTCC |  |
| *ppg1* R-F | GCATCAGTGCCTCCTCTCAGACGTTAGATGATGGGCAAA |  |
| *ppg1* R-R | GAGGAAGGGTGTTAGAGTTA |  |
| *ppg1* C-F | TTTCCCGCTCCCTATCCC |  |
| *ppg1* C-R | CCACCGACAAGCCCGTTT |  |
| *ppg1* O-F | GGGTTCTGTCCGTCCTTA |  |
| *ppg1* O-R | CCGTCCATCCACTCCAT |  |
| *sitA* C-AF | AGGCGGTTACGGGACAC | *sitA* complementation |
| *sitA* C-AR | GGGTGAAGAGCATTGTTTGAGGCGCGGGTTTCGCTTACTG |  |
| *sitA* C-BF | GCATCAGTGCCTCCTCTCAGACGACGGATGATAGGCACG |  |
| *sitA* C-BR | TGAAAGGTAATCGCAGAAC |  |
| *sitA* C-CF | AGGCGGTTACGGGACAC |  |
| *sitA* C-CR | TCGGCGGTTTATAGTGA |  |
| *ppg1* C-AF | AGTCTTTGGGCGTGGTG | *ppg1* complementation  *ppg1* complementation |
| *ppg1* C-AR | GGGTGAAGAGCATTGTTTGAGGCGGATTTATAGCTCGTGG |  |
| *ppg1* C-BF | GCATCAGTGCCTCCTCTCAGACTGTTAGATGATGGGCAAA |  |
| *ppg1* C-BR | CACCACCACTGGACTTTAC |  |
| *ppg1* C-CF | ATTTCCCGCTCCCTATC |  |
| *ppg1* C-CR | CAGTCTGCGGTCGTAAT |  |
| *sitA* P-AR | ACAAAGATGCAAGAGCGGCTCATCAGGCAGGGTCAGAACGGTCA | *sitA/ppg1* deletion |
| *sitA* P-BF | CCCGTAATCAATTGCCCATTGCGTTCGGAGAAACCGTTCA |  |
| *ptr*-F | GATGAGCCGCTCTTGCATCTTTGT | *sitA/ppg1* deletion |
| *ptr*-R | CAATGGGCAATTGATTACGGG |  |
| *nem1* L-F | CGTCGATCTGATCCACCAAGT | *nem1* deletion |
| *nem1* L-R | GGGTGAAGAGCATTGTTTGTGAGATTGCAATGGGATACA |  |
| *nem1* R-F | GCATCAGTGCCTCCTCTCAGAGGCTCCCAAATAGAATTCATG |  |
| *nem1* R-R | CTCACCCCACCTTGTTGATT |  |
| *nem1* C-F | ACTGAACATGGAGATCGGTG |  |
| *nem1* C-R | GAACGCAACACAGCTCAAGG |  |
| *nem1* O-F | CCATGTCCGATTGTTCCTAC |  |
| *nem1* O-R | CGTACGTCGGTCACATATTG |  |
| *spo7* L-F | AGTGTCAGTCACTGAGATGG | *spo7* deletion |
| *spo7* L-R | GGGTGAAGAGCATTGTTTGGTCCGCGACAGTAGTAGAGG |  |
| *spo7* R-F | GCATCAGTGCCTCCTCTCAGAGCTTTGAATCTATACCCCGG |  |
| *spo7* R-R | CACATTGTTACCACCTACTC |  |
| *spo7* C-F | TTGTATCGGGCTGGACATCC |  |
| *spo7* C-R | AGTCAGAGGAGGACTCTGAT |  |
| *spo7* O-F | TCACCTGGTTAAAGGGTCAC |  |
| *spo7* O-R | AGTCGTCGGAAGAGAAAGAC |  |
| *pyrG*-F | GCCTCAAACAATGCTCTTCACCC |  |
| *pyrG*-R | GTCTGAGAGGAGGCACTGATGC |  |
| *pyrG*801-R | CAGGAGTTCTCGGGTTGTCG |  |
| *pyrG*1020-F | ATCGGCAATACCGTCCAGAAGC |  |
| *brlA*-F | GCCTCCAGCGTCAACCTTC | qRT-PCR for genes related to conidiation |
| *brlA*-R | TCTCTTCAAATGCTCTTGCCTC |  |
| *abaA*-F | TCTTCGGTTGATGGATGATTTC |  |
| *abaA*-R | CCGTTGGGAGGCTGGGT |  |
| *nsdC*-F | GCCAGACTTGCCAATCAC | qRT-PCR for genes related to conidiation |
| *nsdC*-R | AGAACGCTGGGTCTGGTGC |  |
| *nsdD*-F | GGACTTGCGGGTCGTGCTA |  |
| *nsdD*-R | AGAACGCTGGGTCTGGTGC |  |
| *sclR*-F | CAATGAGCCTATGGGAGTGG |  |
| *sclR*-R | ATCTTCGCCCGAGTGGTT |  |
| *aflC*-F | ACCATCGTCCCATTCTTG | qRT-PCR for genes related to aflatoxin biosynthesis  qRT-PCR for genes related to aflatoxin biosynthesis |
| *aflC*-R | TCGGTGGTCGGTGATTCAG |  |
| *aflQ*-F | GTCGCATATGCCCCGGTCGG |  |
| *aflQ*-R | GGCAACCAGTCGGGTTCCGG |  |
| *aflR*-F | AAAGCACCCTGTCTTCCCTAAC |  |
| *aflR*-R | GAAGAGGTGGGTCAGTGTTTGTAG |  |
| *aflS*-F | GCTCAGACTGACCGCCGCTC |  |
| *aflS*-R | GCTCAGACTGACCGCCGCTC |  |
| *aflO*-F | GATTGGGATGTGGTCATGCGATT |  |
| *aflO*-R | GCCTGGGTCCGAAGAATGC |  |
| *aflP*-F | ACGAAGCCACTGGTAGAGGAGATG |  |
| *aflP*-R | GTGAATGACGGCAGGCAGGT |  |
| *chsA*-F | ACATTGCGCCAGAAACTGTT | qRT-PCR for genes related to chitin biosynthesis |
| *chsA*-R | CCACGATCTTCTTCCATGCG |  |
| *chsD*-F | TTGGTGCGAGACTTGTGCG |  |
| *chsD*-R | ACGTAGAAGGTAAATGCGATAGCTG |  |
| *chsE*-F | CGATCTTCCTCGCTATGGGT |  |
| *chsE*-R | ACCAAAACGCCTCTCTAGCT |  |
| *actin*-F | ACGGTGTCGTCACAAACTGG | qRT-PCR for endogenous standard gene |
| *actin*-R | CGGTTGGACTTAGGGTTGATAG |  |

Supplementary file references:

Chang PK, Scharfenstein LL, Wei Q, Bhatnagar D. Development and refinement of a

high-efficiency gene-targeting system for *Aspergillus flavus*. J Microbiol Methods.

2010; 81(3): 240-6.
