# Supplementary material for: The target of rapamycin signaling pathway regulates vegetative development, aflatoxin biosynthesis, and pathogenicity in Aspergillus flavus
Source: eLife. 2024 Jul 11;12:RP89478. doi: 10.7554/eLife.89478 (PMC11239180; doi:10.7554/eLife.89478)
Supplement: Figure 2—figure supplement 1—source data 1. [file elife-89478-fig2-figsupp1-data1.zip › Figure 2figure supplement 1 source data 28.zip!/Figure 2-figure supplement 1 -source data 28/Figure 2-figure supplement 1 -source data 28.pdf]

I

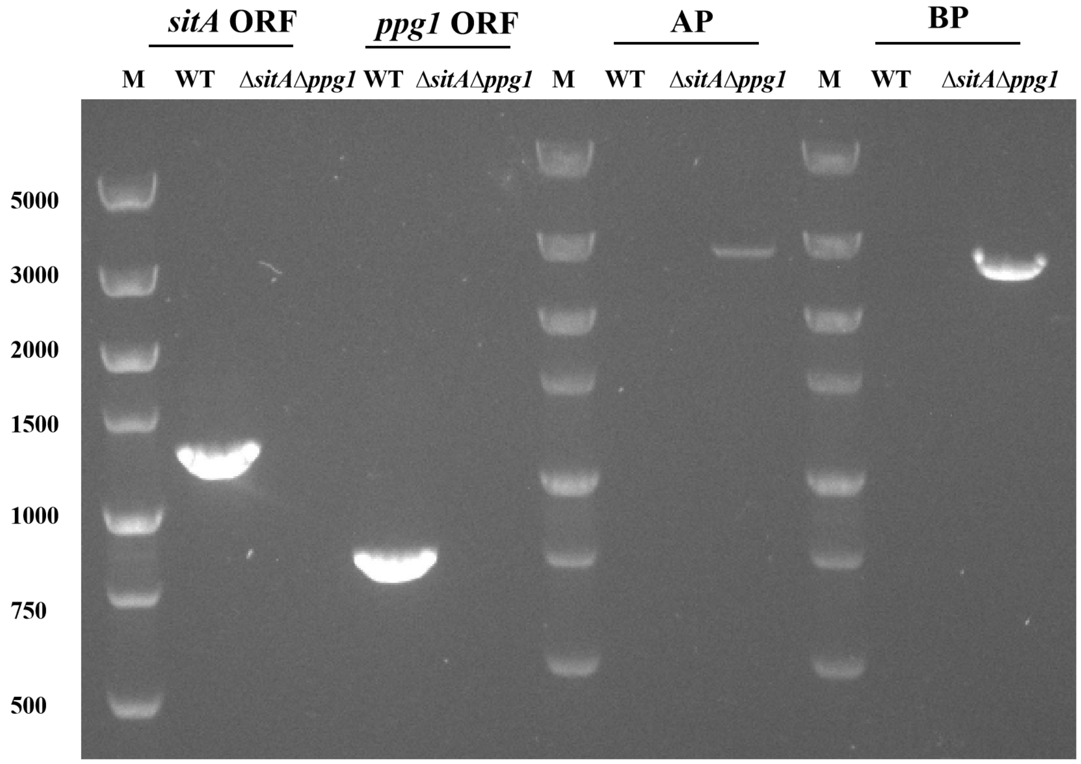

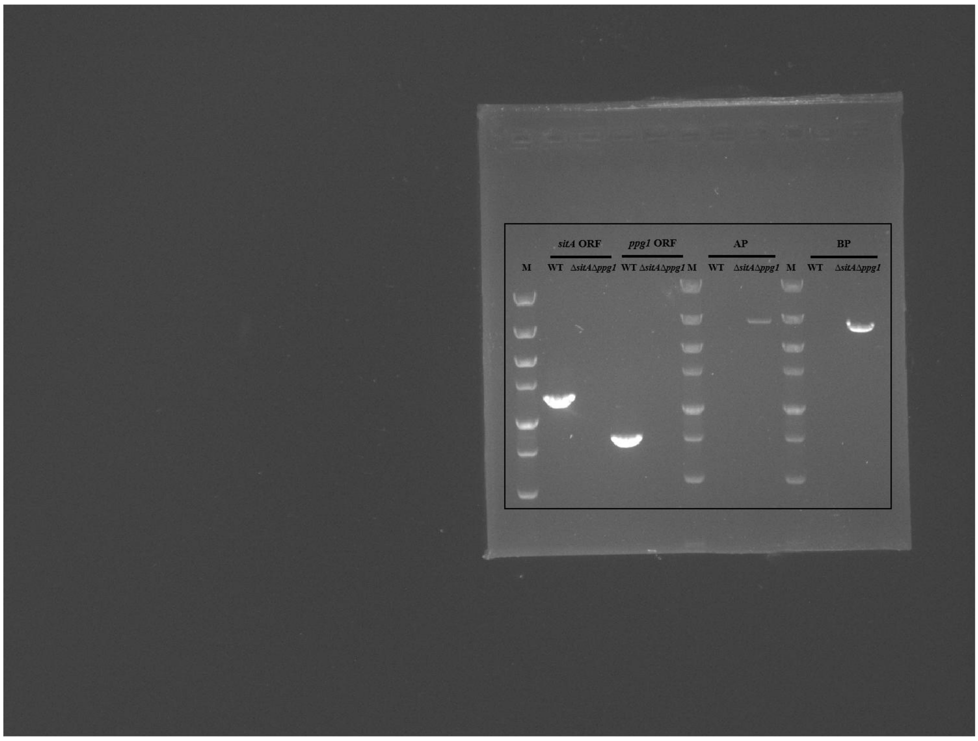

Figure 2-figure supplement 1
